# Associations of Antihypertensive Medication Consumption and Drug-Drug Interaction with Statin and Metformin with Reduced Alzheimer's Disease and Related Dementias Risk among Hypertensive Patients with Mild Cognitive Impairment using High Volume Claims Data

**DOI:** 10.21203/rs.3.rs-2629005/v1

**Published:** 2023-04-13

**Authors:** Sori K. Lundin, Xinyue Hu, Jingna Feng, Karl K. Lundin, Yong Chen, Cui Tao

**Affiliations:** 1 Department of Biostatistics and Data Science, School of Public Health, The University of Texas Health Science Center at Houston, Houston, TX, USA 77030; 2 School of Biomedical Informatics, The University of Texas Health Science Center at Houston, Houston, TX, USA 77030; 3 Center for Biomedical Semantics and Data Intelligence (BSDI), University of Texas Health Science Center at Houston, Houston, TX, USA 77030; 4 Departments of Medicine and Pediatrics, Baylor College of Medicine, Houston, TX, USA 77030; 5 Department of Biostatistics, Epidemiology and Informatics, the Perelman School of Medicine, University of Pennsylvania, Philadelphia, PA, USA 19104

**Keywords:** Alzheimer’s Disease and Related Dementias, Antihypertensives, Drug Repurposing, Drug-drug interaction, Hypertension, Metformin, Statin

## Abstract

**Background:**

While hypertension is a modifiable risk factor of Alzheimer's disease and related dementias (ADRD), limited studies have been conducted on the effectiveness of antihypertensive medications (AHMs) in altering the progression from mild cognitive impairment (MCI) to ADRD; similarly, few studies have assessed drug-drug interactions of AHMs with drugs targeted to modify other risk factors of ADRD such as type II diabetes and hypercholesterolemia.

**Method:**

128,683 unique hypertensive patients with MCI on US-based Optum claims data were identified. Diuretics, beta blockers (BBs), calcium channel blockers (CCBs), angiotensin-converting enzyme inhibitors (ACE inhibitors), and angiotensin II receptor antagonists (ARBs) were identified as five major AHM classes. Baseline characteristics were compared. Cox proportional hazards (PH) models were used to study the association between specific AHM exposure and the progression from MCI to ADRD while controlling for demographic variables, comorbidities, and the use of Statins and Metformin. To examine the association of AHM-Statin or AHM-Metformin interaction with ADRD progression, we also investigated models controlling for the aforementioned confounders, as well as drug-drug interactions.

**Result:**

The study included 100,678 patients who were taking at least one class of AHM and 28,005 who were not taking any AHMs during the study period. AHM users had a higher incidence of comorbidities (all *P*≤0.039) and consumption of Metformin and Statins (both *P*<0.001) compared to non-users. Users of each major AHM class showed significantly lower risk of developing ADRD compared to non-users of that specific drug class (adjusted hazard ratio (aHR): 0.96-0.98; all *P*≤0.048). Within patients on monotherapy (using only one AHM drug), no specific AHM class had significantly lower risk of ADRD diagnosis compared to other AHM drug classes (aHR: 0.97-1.11; all *P*≥0.053). Use of Diuretics or CCBs in combination with Metformin consumption (aHR: 0.89, 0.91, respectively) showed lower risk of MCI to ADRD progression than use without Metformin consumption (aHR: 0.97, 0.98, respectively), whereas use of any of the five major AHMs with Statin consumption (aHR: 0.91-0.94) all showed lower risk than without Statin consumption (aHR: 0.98-1.04).

**Conclusion:**

All five major AHM classes showed a protective effect against ADRD progression among hypertensive patients with MCI. Also, certain combinations of AHMs with Metformin or Statins showed a stronger protective effect compared to AHMs alone, and some drug-drug interactions of AHM-Metformin or AHM-Statin also showed protective effects against progression from MCI to ADRD.

## Introduction

Alzheimer’s disease (AD), the most common cause of dementia, is a major public health burden globally and in the United States. In 2022, an estimated 10.7% of people aged 65 or older had Alzheimer’s dementia in the United States, amounting to 6.5 million Americans. (“2022 Alzheimer’s Disease Facts and Figures,” 2022) Alzheimer's disease and related dementias (ADRD) accounted for nearly 10% of all deaths in the United States in 2019. (Rajan et al., 2021) ADRD was the sixth-leading cause of death in the United States in 2019 (Xu et al., 2020) and the seventh-leading cause of death during the COVID-19 pandemic in 2020 and 2021 (CDC WONDER online database: About Underlying Cause of Death, 1999-2019: https://wonder.cdc.gov/ucd-icd10.html), after COVID-19 entered as the third-leading cause of death. Between 2000 and 2019, deaths from stroke, heart disease and HIV decreased, whereas reported deaths from AD increased more than 145%. (Tejada-Vera, 2013; CDC WONDER online database: About Underlying Cause of Death, 1999-2019 : https://wonder.cdc.gov/ucd-icd.10.html). Its mortality is projected to see an even steeper increase as the population ages, (CDC. “About Alzheimer's disease” : https://www.cdc.gov/aging/publications/aag/alzheimers.html: “Focus on Alzheimer's disease and related dementias” : https://www.ninds.nih.gov/current-research/focus-disorders/focus-alzheimers-disease-and-related-dementias) lending urgency to the search for effective therapies that can treat or prevent ADRD and alleviate the heavy burden this illness places on patients, caregivers, and society. ;

Modern AD research began in the 1970s and has mainly focused on two major hypotheses for the etiology of AD: the cholinergic and amyloid-β (Aβ) hypotheses. (Breijyeh & Karaman, 2020; Tejada-Vera, 2013) Following the isolation and decoding of the protein composition of neurofibrillary tangles from AD-affected brains in 1974 (Iqbal et al., 1974), Davies et al. found evidence of a central cholinergic deficit in AD. (Davies & Maloney, 1976; Iqbal et al., 1974) The discovery of amyloid β peptides as the major components of amyloid plaques, the cloning of APP gene in chromosome 21, and the identification of its mutations in hereditary amyloidosis in 1980s led to the formulation of the amyloid cascade hypothesis. (Hardy & Higgins, 1992) However, despite the heavy burden of ADRD and extensive investment in research on its anatomy, pathogenesis, and neural mechanisms, there have been no medical breakthroughs to prevent or stop the progression of this debilitating cause of dementia to date. (“Focus on Alzheimer's disease and related dementias” : https://www.ninds.nih.gov/current-research/focus-disorders/focus-alzheimers-disease-and-related-dementias) Currently, the few drugs available to treat dementia, cholinesterase inhibitors and memantine, (O’Brien et al., 2017) show clinically marginal efficacy in measures of cognition and global assessment of dementia and no evidence of efficacy in some forms of dementia. (O’Brien et al., 2017; Raina et al., 2008; Stinton et al., 2015) Recently, many investigators have raised questions regarding the cholinergic and Aβ hypotheses and have shifted their research targets to non-amyloid targets, such as tau, neuroinflammation, and neuroprotection. (Cummings et al., 2020; Iqbal et al., 2016) Additionally, there is growing awareness of the need for research identifying modifiable risk factors surrounding development and progression of ADRD. (Edwards et al., 2019; Litke et al., 2021)

Mild Cognitive Impairment (MCI) is a condition that is characterized as an early stage of memory loss or other cognitive ability loss in individuals who maintain the ability to independently perform most activities of daily living. Approximately 12% to 18% of people aged 60 or older are living with MCI. (“Mild cognitive impairment (MCI). Alzheimer's association” : https://www.alz.org/media/Documents/alzheimers-dementia-mild-cognitive-impairment.pdf) While some individuals with MCI bounce back to normal cognition or do not experience further cognitive decline, about 10% to 15% develop dementia each year (“2022 Alzheimer’s Disease Facts and Figures,” 2022; Petersen et al., 2018) and one-third develop dementia due to Alzheimer’s within five years. (Ward et al., 2013) Individuals with MCI are suggested to have 3 to 5 times higher likelihood to progress from MCI to any form of dementia compared to those with normal cognition. (Petersen et al., 1999, 2009; Yaffe et al., 2006)

Hypertension has been suggested as a potential modifiable risk factor for ADRD by a growing body of literature. (“2022 Alzheimer’s Disease Facts and Figures,” 2022; Iadecola et al., 2016; Livingston et al., 2020; Omura et al., 2022; “National Plan to Address Alzheimer’s Disease: 2021 update. Washington, DC: US Department of Health and Human Services; 2021” : https://aspe.hhs.gov/sites/default/files/documents/59cefdd628581b48b2e389891a675af0/napa-national-plan-2022-update.pdf) Managing hypertension has been reported to be a crucial dementia risk reduction strategy by multiple reports, including National Academies of Sciences, Engineering, and Medicine and the Lancet Commission. (Livingston et al., 2020; National Academies of Sciences, Engineering, and Medicine et al., n.d.) There are mixed findings from observational studies about the benefit of lowering BP in late-life to reduce the risk of dementia. (Iadecola et al., 2016) However, various studies including randomized controlled trials (RCTs) and meta-analyses have shown that lowering high blood pressure through the consumption of antihypertensive medications (AHMs) has associations with less risk of ADRD and MCI. (Ding et al., 2020; Levi Marpillat et al., 2013; Moll van Charante et al., 2016; Peters et al., 2008; Rouch et al., 2015; SPRINT MIND Investigators for the SPRINT Research Group et al., 2019) Experimental data suggests that several commonly used AHM drugs may have direct neuroprotective properties, (Hernandorena et al., 2017) and the effect on dementia risk of specific AHMs has been studied with mixed results. Extant observational single cohort studies have identified differing AHM classes as the strongest candidate for prevention. (Barthold et al., 2018; Khachaturian et al., 2006; N.-C. Li et al., 2010)

There are several limitations in the current studies on investigating the effectiveness of antihypertensive medications (AHMs) in reducing the risk of ADRD. Studies on this topic have been inconsistent on source populations, prevalence of confounding factors, study design and analytical methods used, making results difficult to compare. Clinical trials mostly investigate drug efficacy on cognition-related brain outcomes as a secondary or tertiary aim, and therefore are not optimally designed to detect differences in the cognitive/dementia endpoints. (Iadecola et al., 2016) Meta-analysis of community-based cohort, prospective cohort or randomized controlled trial (RCT) studies have found evidence to support an association between the use of antihypertensive medication and lower ADRD or dementia risk. (Ding et al., 2020; Harris et al., 2007; Hofman et al., 2007; Investigators & THE ARIC INVESTIGATORS, 1989) However, there are no large-scale, retrospective cohort studies with sufficient follow up and well-phenotyped individuals with underlying MCI using claims data to investigate whether specific or combinations of AHMs were protective against ADRD risk. In addition, to date, there is no sufficient evidence to recommend prescribing certain classes of antihypertensives for treatment or prevention of ADRD. (Iadecola et al., 2016; Marcum et al., 2022; National Academies of Sciences, Engineering, and Medicine et al., n.d.)

There have been epidemiological and neuropathological studies on identifying potential outstanding modifiable risk factors of ADRD such as type II diabetes mellitus (X. Li et al., 2015; Sun et al., 2020) and hypercholesterolemia (high cholesterol) (Feringa & van der Kant, 2021; Kivipelto et al., 2002; Launer et al., 2001; Varma et al., 2021), as well as studies using anti-diabetic (Herath et al., 2016; Moore et al., 2013; Orkaby et al., 2017; Samaras et al., 2020) or lipid-lowering drugs (Chang et al., 2019; Jick et al., 2000; Lee et al., 2020; Torrandell-Haro et al., 2020); (Zingel et al., 2021); (Galatti et al., 2006; King et al., 2003; Padala et al., 2006) to reduce the risk of ADRD, with mixed results. Recent studies have evaluated the combined effect of these drugs along with AHMs on cognitive decline. Specifically, there have been findings that suggest a potential protective additive effect of AHMs and Statins against ADRD progression. (Barthold et al., 2020) However, the interactive effects of AHMs and lipid-lowering medications (i.e. Statins) or anti-diabetic medications (i.e. Metformin) have not been well studied in relation to ADRD risk.

The aforementioned evidence and limitations led us to propose a new approach to studying the efficacy of fighting ADRD using AHMs. Using claims data, we conducted survival analysis to examine associations between the consumption of antihypertensive medication and ADRD progression in hypertensive patients with MCI. Using this analysis, we compared protective effects of AHM drug classes against ADRD risk. We also examined the additive and interactive effects of generic AHMs and statins, or generic AHMs and metformin in relation to ADRD risk. Lastly, we investigated the semi-competing risk of ADRD according to AHM consumption with death as the semi-competing event.

In this study, we have found that all five major AHM classes showed a protective effect against ADRD progression among hypertensive patients with MCI. Also, AHMs combined with the consumption of Metformin or a Statin demonstrated a stronger protective effect compared to that of AHMs alone. Through our survival analysis, we created one of the first large-scale studies on drug efficacy against cognitive decline endpoints such as ADRD and associations of potential drug-drug interactions with ADRD-related outcomes. We believe this study and analysis pipeline can serve as a template for further, similar large-scale studies in this much needed area of research.

## Methods

### Study population

The US-based Optum de-identified Optum Clinformatics^®^ Data Mart (Optum CDM) claims date of death (DOD) dataset (version 1015; from Jan 1, 2007, to Oct 15, 2020) was used in this study. This dataset is a de-identified database derived from a large, adjudicated claims data warehouse. Patients with one year (twelve consecutive months) or more of claims records without re-enrollment were included in this study. We define the index date as the date of the first ever Mild Cognitive Impairment (MCI) diagnosis of a patient. We used claims data from the Optum CDM database to identify hypertensive patients with a diagnosis of Mild Cognitive Impairment (MCI) who did not have any diagnosis of ADRD prior to the index date. To be eligible for the study, patients must have received inpatient or outpatient care for their MCI diagnosis and have at least one year of prescription records prior to the MCI diagnosis. We excluded patients who only had a single encounter during the enrollment window, as well as those without any diagnostic, procedural, lab, or demographic information prior to the MCI diagnosis. A total of 128,683 unique patients were included in the study.

### Identification of MCI, ADRD, and hypertension

All diagnoses of disease were identified based on the World Health Organization (WHO) International Classification of Diseases, Ninth Revision (ICD-9), (“World Health Organization. (1978). International classification of diseases : [9th] ninth revision, basic tabulation list with alphabetic index.”: https://apps.who.int/iris/handle/10665/39473) Tenth Revision (ICD-10), (“World Health Organization. (2019). International statistical classification of diseases and related health problems (10th ed.).”: https://icd.who.int/browse10/2019/en) and the Anatomical Therapeutic Chemical (ATC) classification system from the WHO Collaborating Centre for Drug Statistics Methodology (WHOCC). (“WHO Collaborating Centre for Drug Statistics Methodology, ATC classification index with DDDs, 2023” : https://www.whocc.no/atc_ddd_index/)) The diagnosis date of incident mild cognitive impairment (ICD-9 331.81, 294.9; ICD-10: G31.83, F09) was identified based on Duan et al., (Bukhbinder et al., 2022; Duan et al., 2020) and the diagnosis date of incident Alzheimer’s disease and related dementias include: the diagnosis date of incident 1) vascular dementia (ICD-9: 290.4; ICD-10: F01.5), non-specific dementia (ICD-9: 290.0, 290.1, 290.2, 290.3, 290.8, 290.9, 797.0; ICD-10: F02.8, F03.9, F04, F05, F06.0, F06.1, F06.2, F06.3, R41.81), lewy-body associated dementia (ICD-9: 331.82; ICD-10: G31.83), frontotemporal dementia (ICD-9: 331.1; ICD-10: G31.0), or Alzheimer’s disease (ICD-9: 331.0; ICD-10: G30.0, G30.1, G30.8, G30.9), (Halpern et al., 2019) or second date of the two 2) pharmacy claim records of anti-dementia drugs (ATC: N06D) within any 6-month period during the enrollment period. (“WHO Collaborating Centre for Drug Statistics Methodology, ATC classification index with DDDs, 2023” : https://www.whocc.no/atc_ddd_index/) Hypertension (ICD-9: 491.x, 492.x, 496; ICD-10: J41.x, J42, J43.2, J43.8, J43.9, J44.x) is defined as having two or more outpatient/inpatient diagnosis of elevated blood pressure on different days within the same 24-month in follow-up period. (Bukhbinder et al., 2022)

### Antihypertensive medications (AHMs)

Our study classified individual drugs according to the WHOCC ATC classification system, (“WHO Collaborating Centre for Drug Statistics Methodology, ATC classification index with DDDs, 2023” : https://www.whocc.no/atc_ddd_index/) and ascertained the classification with domain experts. We examined five major AHM drug classes: diuretics (ATC: C03), beta blockers (ATC: C07), calcium channel blockers (CCBs, including long- and short-acting preparations; ATC: C08), angiotensin-converting enzyme inhibitors (ACE inhibitors; ATC: C09A, C09B), and angiotensin II receptor antagonist (ARBs; ATC: C09C, C09D). Other rarely prescribed drugs such as antiadrenergic agents were classified into Other’ (ATC: C02). Each class includes individual drugs as well as combination drugs (e.g., metoprolol/hydrochlorothiazide included in beta blockers). Mapping of ATC-classified drugs to generic drug names in the OPTUM dataset can be found in **Supplementary Table 1**.

### Confounding factors

Confounding factors were identified by adapting and modifying the confounding factors by Bukhbinder et al. (Bukhbinder et al., 2022) The confounders are 1) demographic information: age in 6 categories (<65, 65-69, 70-74, 75-79, 80-84, 85-90) (age of patients was capped at 90 years), sex, and race, 2) prevalence of comorbidities: atrial fibrillation (AF), congestive heart failure (CHF), chronic obstructive pulmonary disease (COPD), hyperlipidemia (HL), ischemic heart disease (IHD), obesity, traumatic brain injury (TBI), type II diabetes mellitus (T2DM), stroke, anxiety disorder, depression, substance use disorder, and tobacco use, and 3) drug use: statin and metformin use. There was no missing data on demographic information. For comorbidities, ICD-9 and ICD-10 codes were used to identify diseases or medical conditions, except for tobacco use and consumption of statins and metformin, which also used Current Procedural Terminology (CPT) codes and Healthcare Common Procedure Coding System (HCPCS) codes, as well as ATC codes where applicable. Details on each confounding factor can be found in **Supplementary Table 2**.

### Study design

In our study, we examined the association of antihypertensive medications and ADRD progression among the aforementioned population in the ‘Study Population’ section. Prior studies indicate that AHMs have no to little effect on ADRD disease progression in normotensive patients, (Ding et al., 2020; Lonn et al., 2016) and including normotensive patients with MCI to our population would add a potential confounder (hypertension status) that could not be well controlled as very few normotensive patients are taking AHMs. Therefore, to better focus on the target population, the study population was narrowed down from patients with Mild Cognitive Impairment (MCI) to hypertensive patients with MCI. The index date is defined as the date of the first MCI diagnosis, and time to follow-up (or time at risk) is defined as either 1) the time elapsed from the index date to the first date of incident Alzheimer's and Related Dementias (ADRD) diagnosis if the patient is diagnosed with ADRD, or 2) the time elapsed from the index date to the date of the last healthcare encounter, including any healthcare encounters regardless of the length of stay or outpatient records with one or more ICD codes.

Consumption of antihypertensive medication is classified as follows: ‘AHM users’ refer to patients consuming at least one antihypertensive medication according to the WHOCC ATC classification system, whereas ‘AHM non-users’ refer to patients who do not take any form of antihypertensive medications. ‘Specific AHM users’ (e.g., ‘ARB users’), refer to patients consuming AHMs including but not limited to a single or combination of ARBs, and ‘ARB non-users’ refer to patients that do not take any ARBs, but may or may not consume AHMs from other drug classes. In a subgroup analysis of patients that only take one AHM, ‘ARB only users’ refer to patients who use ARBs as a monotherapy and ‘ARB only non-users’ are defined as patients who consume one AHM from some other drug class other than ARBs as a monotherapy.

### Statistical analyses

#### Analytical approach

Baseline characteristics were compared between groups by the Chi-square test for categorical variables and the Kruskal-Wallis test for continuous variables. For descriptive statistics, the baseline characteristics were reported by number and percentages (%) for categorical variables and mean and 95% confidence interval (CI) for continuous variables. We also used Chi-square and Kruskal-Wallis tests to determine the independent associations between the ADRD diagnosis status and AHM consumption.

The Cox proportional hazards (PH) model was used to study the association between AHM exposure and the time from MCI to ADRD, after adjusting for the aforementioned confounders. To examine hazard ratio proportionality assumptions, a visual assessment of Kaplan-Meier plots and log(−log) plots were used. (Hess, 1995) In addition, we evaluated the violation of proportionality assumption via testing procedure based on scaled Schoenfeld residuals. (Schoenfeld, 1982) As the proportionality assumption was not violated, we used the traditional log-rank test. We evaluated the cumulative incidence and the effects of the AHM consumption using the semi-competing risks model i.e., Illness-Death model, with death (terminal event) without the ADRD diagnosis (non-terminal event) as a competing event. (Peng & Fine, 2007; Putter et al., 2007; Xu et al., 2010) We also performed sensitivity analysis among hypertensive patients with MCI without any Statin or Metformin consumption to evaluate changes in association between AHM exposure and the progression from MCI to ADRD, which did not show any significant difference.

All analyses were performed in RStudio with R version 4.1.2. A two-tailed *P* ≤ 0.05 was considered statistically significant.

## Results

### Baseline characteristics

A total of 128,683 hypertensive patients with Mild Cognitive Impairment were included in the analysis, of which 100,678 were using at least one antihypertensive medication and 28,005 not using any. As shown in Table 1, more than half of the AHM users (58.7%) were female and AHM users had a higher ratio of females than non-users (*P*<0.001). Hypertensive patients with MCI were primarily white in their race/ethnicity, accounting for 70.6% of AHM users and 78.1% of non-users. AHM users had significantly younger mean age at first MCI diagnosis than that of non-users (Mean (95% CI): AHM users 75.53 (75.47, 75.59), AHM non-users: 77.95 (77.85, 78.05); *P* < 0.001), and significantly longer mean time to ADRD from MCI diagnosis in months (AHM users: 22.87 (22.71, 23.04), AHM non-users: 21.96 (21.68, 22.23); *P* < 0.0001). Ratio of deceased patients and time to death from MCI diagnosis did not have any significant difference between AHM users and non-users (both *P* ≥ 0.113). AHM users consistently had higher incidence for all the comorbidities (all *P* ≤ 0.039) and consumption of metformin and statins (both *P* < 0.001) compared to AHM non-users.

Table 2 displays consumption of each AHM class and major individual or combination AHMs among the patients by their incident ADRD diagnosis. Among 71,558 patients who were diagnosed with ADRD, the majority (78.2%) of them were taking at least one antihypertensive medication (any AHMs) but did not show significant difference in consumption compared to 57,125 patients without ADRD (non-ADRD: 78.3%; *P* = 0.781). ADRD patients consuming Diuretics, Beta-blockers, CCBs, ARBs and Other AHMs all had significantly higher ratio of consumption than non-ADRD patients (all *P* < 0.001), whereas consumption of ACE inhibitors were not significantly different between patients with or without ADRD (*P* = 0.288).

### Longitudinal Analysis

#### ADRD risk based on AHM use

Among hypertensive patients with MCI, users of all five major AHM classes except ‘Other AHMs’ (*P* = 0.199) showed significantly lower risk of developing ADRD compared to non-users that do not take any AHMs from the specific drug class, but may or may not consume AHMs from other drug classes. Diuretics users had 4% lower risk of ADRD risk than non-users (95% CI: (0.95, 0.98); *P* < 0.001). Users of Beta Blockers, CCBs, ACE inhibitors, and ARBs also had lower risk of progression to ADRD from MCI and hypertension (adjusted hazard ratio (aHR) (95% CI): Beta Blockers 0.98 (0.96, 1.00), *P* = 0.023; CCBs 0.97 (0.95, 0.98), *P* < 0.001; ACE inhibitors 0.98 (0.97, 1.00), *P* = 0.048; ARBs 0.98 (0.97, 1.00), *P* = 0.046). Patients who use any form of AHMs had 6% higher adjusted hazard ratio of ADRD (95% CI: (1.04, 1.08), *P* < 0.001) than non-users (Table 3).

We also performed subgroup analysis to compare ADRD risk between patients on monotherapy (using only one AHM drug). None of the consumption of specific AHM class had any significantly lower risk of ADRD diagnosis compared to that of other AHM drug classes (*P* = 0.053 - 0.974). More details on Cox PH analysis of AHM consumption associated with ADRD risk are displayed in Table 3.

#### Drug-Drug Interaction Analysis

**Figure 1.**
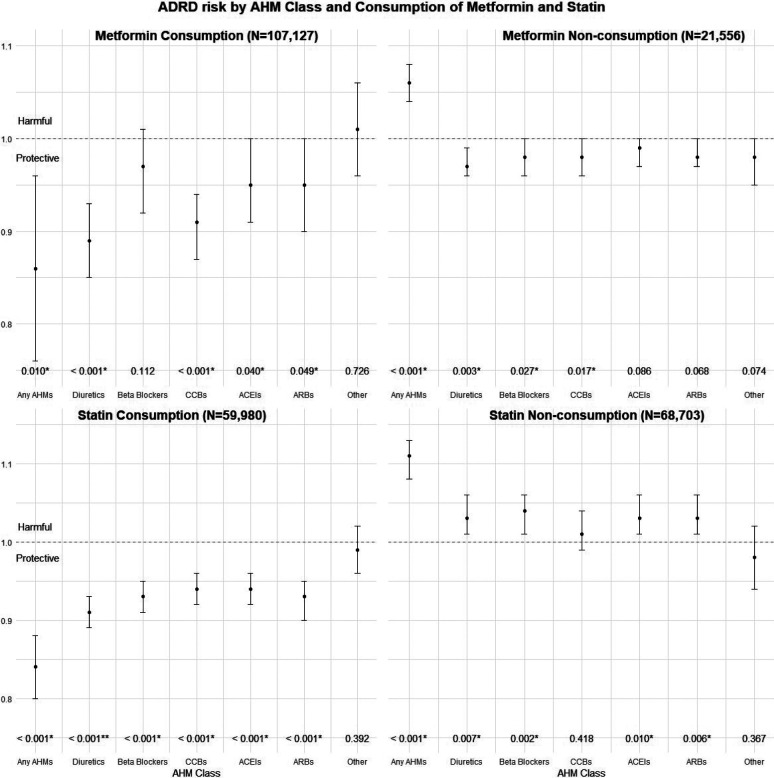
Adjusted Hazard Ratios of ADRD risk by AHM class and consumption of Metformin and Statin

Significant interactions were found between certain AHM classes and the consumption of Metformin in relation to ADRD risk, after adjusting for the aforementioned covariates (Table 4, Fig. 1). Specifically, under Metformin consumption, risk of developing ADRD for any AHM user was significantly lower than that of AHM non-users (aHR (95% CI): 0.86 (0.76, 0.96), *P* = 0.010), whereas under Metformin non-consumption, the hazard was significantly higher (aHR (95% CI): 1.06 (1.04, 1.08), *P* < 0.001). The interaction between any AHMs and Metformin was significant as well (*P* < 0.001). Similarly, the interaction term of Diuretics use and Metformin consumption was also significant (*P* < 0.001), and under consumption of Metformin, Diuretics use showed stronger protective effect of (aHR (95% CI): 0.89 (0.85, 0.93), *P* < 0.001) against ADRD risk than under non-consumption of Metformin (aHR (95% CI): 0.97 (0.96, 0.99), *P* = 0.003). CCBs users had 9% lower risk of MCI to ADRD progression compared to non-users under consumption of Metformin (95% CI: (0.87, 0.94), *P* < 0.001), but 2% lower risk under non-consumption of Metformin (95% CI: (0.96, 1.00), *P* = 0.017).

Table 5 and Fig. 1 show similar analyses for consumption of Statins, where we found significant adjusted interactions between all major AHM classes excluding ‘Other’ and consumption of Statins in relation to ADRD risk (all *P* < 0.001 except for ‘Other’: *P* = 0.575). Under consumption of Statins, use of any AHMs or major AHM classes except for ‘Other’ AHMs showed protective effect against ADRD risk, whereas the use showed harmful effect under non-consumption of Statins. For example, users of Beta Blockers had significantly lower adjusted risk of ADRD risk compared to non-users when they also consume Statin (aHR (95%CI): 0.93 (0.91, 0.95). *P* < 0.001), whereas the risk was significantly higher when they do not consume a Statin (aHR (95% CI): 1.04 (1.01, 1.06), *P* < 0.001).

## Discussion

This is the first study, to the best of our knowledge, to assess and compare the associations between all major antihypertensive medication classes and ADRD risk using large electronic medical records data, such as claims data. We found statistically significant protective effects in relation to ADRD development in five major AHM classes including Diuretics, Beta Blockers, Calcium Channel Blockers, Angiotensin-converting enzyme inhibitors, and angiotensin II receptor blockers. This is in line with previous literature suggesting that AHMs may have associations with reduced ADRD risk. For example, a retrospective cohort study examining the relationship of AHM use with incidence of AD among 5,092 elderly residents aged 65 years and older of Cache County, Utah found that use of any AHM at baseline was associated with reduced incident AD risk (adjusted HR (95% CI): 0.64 (0.41, 0.98)) compared to non-use, and use of Diuretics was associated with a greater reduction in AD risk (adjusted HR (95% CI): 0.57 (0.33, 0.94)). (Khachaturian et al., 2006) In a randomized clinical trial study including 9,361 participants aged 50 years or older in the United States and Puerto Rico, participants in the intense BP treatment group had significantly lower risk of MCI and adjudicated probable dementia (HR (95% CI): 0.85, (0.74, 0.97)). Among 819,491 predominantly male participants aged 65 or more with cardiovascular disease, ARBs had a hazard rate for incident dementia among ARBs users of 0.76 (95% CI: (0.69,0.84)) compared with the cardiovascular comparator and 0.81 (95% CI: (0.73, 0.90)) compared with the lisinopril users. (Khachaturian et al., 2006; N.-C. Li et al., 2010)

Our findings suggest that while all five major AHM classes show a protective effect against ADRD progression among hypertensive patients with MCI, the superiority of specific AHM classes was not demonstrated. Specifically, the results reveal that among the five classes, Diuretics showed the lowest risk of ADRD for users, but adjusted hazard ratios were all comparable (0.96 - 0.98) among all classes, with no clear superiority of one specific AHM class over the others. This was even clearer in subgroup analysis of patients on monotherapy, where none of the drug classes had significantly lower hazard ratios compared to those of other drug classes. Although knowledge on the potential superiority of specific AHM classes in reducing ADRD risk is very limited, our findings are consistent with the result from a meta-analysis of prospective cohort studies on the subject, where it was reported that none of the specific AHM class users with high blood pressure showed a significant pooled hazard ratio (HR) for incident dementia (HR (95% CI): Diuretics 0.95 (0.83, 1.09); Beta Blockers 0.95 (0.83, 1.10); CCBs 1.04 (0.86, 1.24); ACE inhibitors 1.11 (0.96, 1.29); ARBs 0.88 (0.71, 1.09)) or incident AD (HR (95% CI): Diuretics 0.95 (0.77, 1.16); Beta Blockers 1.00 (0.79, 1.29); CCBs 1.05 (0.76, 1.45); ACE inhibitors 1.18 (0.87, 1.60); ARBs 0.79 (0.53, 1.18)) when compared to users of other AHM classes. (Ding et al., 2020) Barthold et al. also reported that renin-angiotensin system (RAS) acting AHMs, which include ACE inhibitors and ARBs, did not show significantly higher protective effects against onset of AD than non-RAS-acting AHMs among females (OR (95% CI): 0.985 (0.963, 1.007)). (Barthold et al., 2018) Since class-level comparisons did not identify any AHM class with higher protective effects against ADRD progression, further analysis on drug-level comparisons may be needed.

To our knowledge, there has been no study that has investigated the drug-drug interactions between AHMs and Statins (Barthold et al., 2020) or between AHMs and Metformin in relation to the risk of ADRD. Our study found that the use of Diuretics or Calcium Channel Blockers in combination with Metformin was associated with 11% and 9% lower risk of ADRD, respectively, as compared to individuals not using these AHMs. On the other hand, the use of these AHMs without Metformin was associated with a 3% and 2% lower risk of ADRD, respectively. Furthermore, our study found that all five major AHMs in combination with Statins have similar protective effects in reduced ADRD risk, with a higher difference in hazard ratios with or without Statin consumption. These findings indicate not only the additive effects of these drug combinations, but also a possible protective role of drug-drug interactions against the progression of ADRD.

This study has a number of limitations. Firstly, we were not able to investigate genetic factors in relation to ADRD risk due to the unavailability of genetic data in claims data. As apolipoprotein E (APOE) genotype is a well-known genetic risk factor for dementia, Alzheimer’s disease, and cardiovascular disease (CVD), (Haan & Mayeda, 2010) linking genetic data from high-volume genetic sequence databases such as GenBank may help account for genetic factors. Also, as the study population is from claims data, the integrity of data relies exclusively on reported claims. For example, although we have access to time-varying changes in prescription, lack of adherence to medications have not been considered, though any non-adherence to medications would generally be expected to skew our study in favor of the null hypothesis. In addition, the dosage of AHM is not accounted for in our study, thereby limiting our ability to study any dose-related effects. Other conditions such as diagnosis of diseases e.g., ADRD, MCI, hypertension or death may not be reported if the patient no longer subscribes to the insurance system. Also, other relevant social determinants of health (SDOH) such as socioeconomic status, access to healthcare, and social support networks are not available on claims data. Therefore, the associations reported in this study should be interpreted with caution and warrant replication in various study designs such as RCTs or prospective cohort studies that would allow overcoming these limitations.

## Conclusion

All five major AHM classes showed a protective effect against ADRD progression among hypertensive patients with MCI. Also, certain combinations of AHMs with use of Metformin or Statins showed a stronger protective effect compared to AHMs alone, and some drug-drug interactions of AHM-Metformin or AHM-Statin also showed protective effects against progression from MCI to ADRD. Replications of this study with different study designs are warranted.

## Figures and Tables

**Table 1.** Demographic information of 128,683 hypertensive patients with mild cognitive impairment according to antihypertensive medication consumption ^a^

	AHM non-users (*N* = 28,005)	AHM users (*N* = 100,678)	*P*-value
**Sex**			
Female	15,940 (56.9%)	59,114 (58.7%)	< 0.001*
Male	12,065 (43.1%)	41,564 (41.3%)	
**Race/Ethnicity**			
Black	1,631 (5.8%)	9,913 (9.9%)	
Asian	462 (1.7%)	2,454 (2.4%)	< 0.001*
Hispanic	1,238 (4.4%)	10,075 (10.0%)	
White	21,857 (78.1%)	71,106 (70.6%)	
Other/Unknown	2,817 (10.1%)	7,130 (7.1%)	
**Age at first MCI diagnosis**	77.95 (77.85, 78.05)	75.53 (75.47, 75.59)	< 0.001*
**Incident ADRD diagnosis**	15,594 (55.7%)	55,964 (55.6%)	0.781
**Time to ADRD from MCI diagnosis (in months)**	21.96 (21.68, 22.23)	22.87 (22.71, 23.04)	< 0.001*
**Death**	9,404 (33.6%)	33,298 (33.1%)	0.113
**Time to death from MCI diagnosis (in months)**	29.25 (28.93, 29.57)	30.87 (30.68, 31.06)	0.159
**Atrial Fibrillation (AF)**	9,076 (32.4%)	35,328 (35.1%)	< 0.001*
**Congestive Heart Failure (CHF)**	9,009 (32.2%)	41,000 (40.7%)	< 0.001*
**Chronic obstructive pulmonary disease (COPD)**	8,298 (29.6%)	37,036 (36.8%)	< 0.001*
**Hyperlipidemia**	25,092 (89.6%)	93,466 (92.8%)	< 0.001*
**Ischemic heart disease (IHD)**	14,191 (50.7%)	58,352 (58.0%)	< 0.001*
**Obesity**	7,310 (26.1%)	36,468 (36.2%)	< 0.001*
**Stroke**	8,628 (30.8%)	36,247 (36.0%)	< 0.001*
**Traumatic brain injury (TBI)**	10,626 (37.9%)	38,998 (38.7%)	0.016*
**Type 2 Diabetes Mellitus (T2DM)**	9,184 (32.8%)	43,279 (43.0%)	< 0.001*
**Anxiety Disorder**	8,000 (28.6%)	28,128 (27.9%)	0.039*
**Depression**	10,015 (35.8%)	37,983 (37.7%)	< 0.001*
**Substance Disorder**	3,556 (12.7%)	20,496 (20.4%)	< 0.001*
**Tobacco use**	12,159 (43.4%)	44,940 (44.6%)	< 0.001*
**Metformin consumption**	582 (2.1%)	20,974 (20.8%)	< 0.001*
**Statin consumption**	3,029 (10.8%)	65,674 (65.2%)	< 0.001*

*Note*: *P* values were estimated by the chi-square test for categorical variables and the Kruskal-Wallis test for continuous variables.

*AHM non-users:* patients who do not take any identified antihypertensive medications according to WHOCC ATC classification system; *AHM users*: patients consuming at least one antihypertensive medication according to WHOCC ATC classification system.

a Data are expressed as mean (95% CI) for continuous variables or number (%) for categorical variables.

* *P* ≤ 0.05 is regarded as statistically significant.

**Table 2.** Consumption of each AHM class and major individual or combination AHMs among 128,683 hypertensive patients with MCI according to incident ADRD diagnosis.

	Non-ADRD (*N* = 57,125)	ADRD (*N* = 71,558)	*P*-value
**Any AHMs**			
Consumption of any AHMs	44,714 (78.3%)	55,964 (78.2%)	0.781
**Diuretics**			
Consumption of any Diuretics	27,228 (47.7%)	35,582 (49.7%)	< 0.001*
Hydrochlorothiazide	13,068 (22.9%)	16,040 (22.4%)	0.050
Chlorothiazide	1,641 (2.9%)	1,605 (2.2%)	< 0.001*
**Beta-blockers**			
Consumption of any Beta-blockers	28,731 (50.3%)	37,396 (52.3%)	< 0.001*
Metoprolol	18,897 (33.1%)	25,442 (35.6%)	< 0.001*
Atenolol	5,983 (10.5%)	8,059 (11.3%)	< 0.001*
Carvedilol	6,888 (12.1%)	8,609 (12.0%)	0.889
Nebivolol	1,474 (2.6%)	1,505 (2.1%)	< 0.001*
**CCBs**			
Consumption of any CCBs	22,007 (38.5%)	28,379 (39.7%)	< 0.001*
Amlodipine	19,787 (34.6%)	25,615 (35.8%)	< 0.001*
**ACE inhibitors**			
Consumption of any ACE inhibitors	30,840 (54.0%)	38,418 (53.7%)	0.288
Enalapril	13,068 (22.9%)	16,040 (22.4%)	0.050
Lisinopril	20,887 (36.6%)	27,106 (37.9%)	< 0.001*
Lisinopril/Diuretics	4,596 (8.1%)	5,055 (7.1%)	< 0.001*
**ARBs**			
Consumption of any ARBs	35,198 (61.6%)	43,366 (60.6%)	< 0.001*
Valsartan	3,971 (7.0%)	4,926 (6.9%)	0.643
Valsartan/Diuretics	2,048 (3.6%)	2,249 (3.1%)	< 0.001*
Olmesartan	2,231 (3.9%)	< 0.001*	< 0.001*
Olmesartan/Diuretics	1,080 (1.9%)	1,210 (1.7%)	0.008*
**Other**			
Consumption of any other AHMs	6,486 (11.4%)	8,543 (11.9%)	0.001*
Prazosin	349 (0.6%)	420 (0.6%)	0.604
Methyldopa	56 (0.1%)	68 (0.1%)	0.935

*Note*: *P* values were estimated by the chi-square test for categorical variables and the Kruskal-Wallis test for continuous variables. Abbreviations: AHM, antihypertensive medication; CCBs, calcium channel blockers; ACE inhibitors, angiotensin converting enzyme inhibitors; ARBs, angiotensin II receptor blockers.

a Data are expressed as mean (95% CI) for continuous variables or number (%) for categorical variables.

* *P* ≤ 0.05 is regarded as statistically significant.

**Table 3.** Cox proportional hazards analysis of AHM consumption associated with ADRD risk ^a^

	Any AHMs		Diuretics		Beta Blockers		CCBs		ACE inhibitors		ARBs		Other	
	aHR (95% CI)	*P*- value	aHR (95% CI)	*P*- value	aHR (95% CI)	*P*- value	aHR 95% CI)	*P*- value	aHR (95% CI)	*P*- value	aHR (95% CI)	*P*- value	aHR (95% CI)	*P*- value
**Users vs. Non-users** ^b^	1.06 (1.04, 1.08)	< 0.001*	0.96 (0.95, 0.98)	< 0.001*	0.98 (0.96, 1.00)	0.023*	0.97 (0.95, 0.98)	< 0.001*	0.98 (0.97, 1.00)	0.048*	0.98 (0.97, 1.00)	0.046*	0.98 (0.96, 1.01)	0.199
**Only users vs. Only non-users** ^c^	N/A	N/A	1.11 (0.99, 1.24)	0.063	1.00 (0.94, 1.06)	0.962	0.97 (0.91, 1.03)	0.301	1.18 (1.00, 1.39)	0.053	0.97 (0.90, 1.05)	0.429	1.00 (0.81, 1.23)	0.974

*Note*: For the definition of ‘users’, ‘non-users’, ‘only users’ and ‘only non-users’, refer to ‘Study design’ in Methods section.

Abbreviations: AHM, antihypertensive medication; CCBs, calcium channel blockers; ACE inhibitors, angiotensin-converting enzyme inhibitors; ARBs, angiotensin II receptor blockers; CI, confidence interval; aHR, adjusted hazard ratio.

a HR was adjusted for age, sex, race, atrial fibrillation, congestive heart failure, chronic obstructive pulmonary disease, hyperlipidemia, ischemic heart disease, obesity, traumatic brain injury, type II diabetes, stroke, anxiety disorder, depression, substance use disorder, and tobacco use, and consumption of metformin and statin.

b Number of users vs non-users (total *N*=128,683): Any AHMs 100,678 vs. 28,005; Diuretics 62,810 vs. 65,873; Beta-blockers 66,127 vs. 62,556; CCBs 50,386 vs. 78,297; ACE inhibitors 69,258 vs. 59,425; ARBs 78,564 vs. 50,119; Other 15,029 vs. 113,654.

c Number of only users vs only non-users (total *N*=9,385): Diuretics 604 vs. 8,781; Beta-blockers 4,396 vs. 4,989; CCBs 2,371 vs. 7,014; ACE inhibitors 280 vs. 9,105; ARBs 1,554 vs. 7,831; Other 180 vs. 9,205.

* *P* ≤ 0.05 is regarded as statistically significant.

**Table 4.** Interaction of consumption of Metformin with AHM classes in relation to ADRD risk based on Cox PH model (*N*=128,683)

AHM Class	Comparison	Consumption of Metformin ^a^	Adjusted HR ^b^ (95% CI)	*P*-value	*P*-value for AHM*Metformin Interaction ^c^
**Any AHMs**	Users vs. Non-users	Y	0.86 (0.76, 0.96)	0.010*	< 0.001*
	Users vs. Non-users	N	1.06 (1.04, 1.08)	< 0.001*	
**Diuretics**	Users vs. Non-users	Y	0.89 (0.85, 0.93)	< 0.001*	< 0.001*
	Users vs. Non-users	N	0.97 (0.96, 0.99)	0.003*	
**Beta Blockers**	Users vs. Non-users	Y	0.97 (0.92, 1.01)	0.112	0.837
	Users vs. Non-users	N	0.98 (0.96, 1.00)	0.027*	
**CCBs**	Users vs. Non-users	Y	0.91 (0.87, 0.94)	< 0.001*	< 0.001*
	Users vs. Non-users	N	0.98 (0.96, 1.00)	0.017*	
**ACEIs**	Users vs. Non-users	Y	0.95 (0.91, 1.00)	0.040*	0.189
	Users vs. Non-users	N	0.99 (0.97, 1.00)	0.086	
**ARBs**	Users vs. Non-users	Y	0.95 (0.90, 1.00)	0.049*	0.127
	Users vs. Non-users	N	0.98 (0.97, 1.00)	0.068	
**Other**	Users vs. Non-users	Y	1.01 (0.96, 1.06)	0.726	0.197
	Users vs. Non-users	N	0.98 (0.95, 1.00)	0.074	

Abbreviations: AHM, antihypertensive medication; CCBs, calcium channel blockers; ACEIs, angiotensin-converting enzyme inhibitors; ARBs, angiotensin II receptor blockers; CI, confidence interval; HR, hazard ratio.

a Consumption of Metformin: Yes (Y) *N* = 107,127; No (N) *N* = 21,556.

b HR was adjusted for age, sex, race, atrial fibrillation, congestive heart failure, chronic obstructive pulmonary disease, hyperlipidemia, ischemic heart disease, obesity, traumatic brain injury, type II diabetes, stroke, anxiety disorder, depression, substance use disorder, and tobacco use, and consumption of statin.

c Model formula: logit(*h*(*t*)/*h*_0_(*t*)) = *β*1(Specific AHM users) + *β*2(Metformin consumption) + *β*3(Specific AHM users*Metformin consumption; adjusted for aforementioned covariates.

* *P* ≤ 0.05 is regarded as statistically significant.

**Table 5.** Interaction of consumption of Statin with AHM classes in relation to ADRD risk based on Cox PH model (*N*=128,683)

AHM Class	Comparison	Consumption of Statin	Adjusted HR (95% CI)	*P*-value	*P*-value for AHM*Statin Interaction
**Any AHMs**	Users vs. Non-users	Y	0.84 (0.80, 0.88)	< 0.001*	< 0.001*
	Users vs. Non-users	N	1.11 (1.08, 1.13)	< 0.001*	
**Diuretics**	Users vs. Non-users	Y	0.91 (0.89, 0.93)	< 0.001*	< 0.001*
	Users vs. Non-users	N	1.03 (1.01, 1.06)	0.007*	
**Beta Blockers**	Users vs. Non-users	Y	0.93 (0.91, 0.95)	< 0.001*	< 0.001*
	Users vs. Non-users	N	1.04 (1.01, 1.06)	0.002*	
**CCBs**	Users vs. Non-users	Y	0.94 (0.92, 0.96)	< 0.001*	< 0.001*
	Users vs. Non-users	N	1.01 (0.99, 1.04)	0.418	
**ACEIs**	Users vs. Non-users	Y	0.94 (0.92, 0.96)	< 0.001*	< 0.001*
	Users vs. Non-users	N	1.03 (1.01, 1.06)	0.010*	
**ARBs**	Users vs. Non-users	Y	0.93 (0.90, 0.95)	< 0.001*	< 0.001*
	Users vs. Non-users	N	1.03 (1.01, 1.06)	0.006*	
**Other**	Users vs. Non-users	Y	0.99 (0.96, 1.02)	0.392	0.575
	Users vs. Non-users	N	0.98 (0.94, 1.02)	0.367	

Abbreviations: AHM, antihypertensive medication; CCBs, calcium channel blockers; ACEIs, angiotensin-converting enzyme inhibitors; ARBs, angiotensin II receptor antagonist; CI, confidence interval; HR, hazard ratio.

a Consumption of Statin: Yes (Y) *N* = 59,980; No (N) *N* = 68,703.

b HR was adjusted for age, sex, race, atrial fibrillation, congestive heart failure, chronic obstructive pulmonary disease, hyperlipidemia, ischemic heart disease, obesity, traumatic brain injury, type II diabetes, stroke, anxiety disorder, depression, substance use disorder, and tobacco use, and consumption of metformin.

c Model formula: logit(*h*(*t*)/*h*_0_(*t*)) = *β*1(Specific AHM users) + *β*2(Statin consumption) + *β*3(Specific AHM users*Statin consumption + *β*(covariates in ^b^).

* *P* ≤ 0.05 is regarded as statistically significant.
